# Collectanea Medica

**Published:** 1819-12

**Authors:** 


### COLLECTANEA MEDICA.

### *Observations on some Points relating to the Physiology and Pathology of the Ear.**

WHEN the ears are stopped, and a watch is brought in contact with any part of the head, face, teeth, or neck; or if a stick, water, &c. be interposed between any of these parts and the watch, the sound will be heard as well as when the ears are open.

It has been supposed, that the sound is mechanically conveyed through the flesh and bone in the same way it is through a macerated bone, piece of wood, &c.; but, if it were so, it must be heard always, when the auditory nerve is perfect, at whatever part of the head, face, &c. the watch is applied: but this is not the case. Where the hearing through the meatus externus has been perfect, and where there has been no apparent alteration in the structure of the head, face, &c. I have seen many who could hear from only one of these parts, and several who could not hear from any of them.

If I stop my ears, and rest my chin on the petrous portion of the temporal bone in a macerated skull, and place my watch in contact with any part of the skull, I can hear the sound perfectly. I saw a boy who was born deaf and dumb, but had been taught to speak; and, when a watch touched the left side of his face, he could hear it; but, when it touched any part of the right side, he could not in the least.

A man who was recovering from an illness had become so deaf of the left ear, that he could just hear my watch when put very near it: he heard perfectly of the right ear. I desired he would stop his ears until he could not hear my watch when put nearly in contact with them: I then let it touch the left side of the face, &c.; he just heard it; but when I let it touch the right side, he heard it distinctly.

If sound is conveyed mechanically through the flesh and bone, what, in these two cases, should hinder it from being heard distinctly when the watch touched either side of the face, any more than in the macerated skull?

If sound is not conveyed mechanically through the head, face, &c. it must be through some other medium; and that I believe to be the porta dura of the seventh pair of nerves, and some other nerves connected with it.

On dissecting the seventh pair of nerves in man, I find, at the bottom of the meatus auditorius internus, a communication between the portio mollis and portio dura.

In the sheep I have observed the same communication.

In fishes, several nerves that have a communication with the auditory nerve, are spread on the skin over the whole head.

If we consider how the portio dura is connected by nervous substance with the portio mollis, its extraordinary course, its receiving the branch of the vidian nerve and the chorda tympani; and, when it has got out of the foramen stylo-mastoideum, its great expansion, I think we may conclude, that it was made to serve some greater purpose than has hitherto been ascribed to it.

---

* By Mr. Swan, Surgeon of Lincoln Hospital. Extracted from the ninth volume of the *Medico-Chirurgical Transactions.*

That this provision of nature has been useful to deaf people, the following case, which may be found in HALLER's *Prælectiones Academicæ*, will prove: "Musicus fuit in aulâ, ex morbo factus surdaster, prehendebat vestibulum mordicus, et tum omnino chelyn ex arte, pulsabat."

That it might be useful to many, could proper instruments be made to increase the effect of sound, and especially to those who are deaf and dumb, if properly persevered in, is, I think, probable; but it must be remembered that, where the disease is in the nerve, no good can be derived from it, which may be ascertained after a few trials, by the expression of the child, if a sounding body is applied to the head, face, neck, or teeth: and that many deaf and dumb can hear in this way, I am myself, from experiment, well convinced.

If, from what has been said, it should appear probable, that sound is conveyed by the portio dura to the portio mollis in man, it will, I think, be reasonable to conclude, that the nerves, which are spread on the soft parts of the heads of fishes, answer in a great measure the same purpose the tympanum does in man; and, though in man this provision is not necessary when the tympanum is perfect, yet, when that is imperfect, it becomes the means of conveying sound to the portio mollis, and thus answers one of the most important purposes in the animal economy.

---

*An Alphabetical List of the Plants and other Articles of the Materia Medica mentioned in the Works of* HIPPOCRATES, *with the Latin Version of* FÖES, *and the Synonyms of* LINNÆUS. [Extracted from a Memoir by Dr. PAULET.]

[*Concluded from page* 389.]

Κριθη et κριθος; hordeum; hordeum vulgare.

Κριθμον; *voyez* κρεθμον.

Κριθος αχιλλοιδες; hordeum achilleum; hordeum hexasticum *ou* H. jubatum.

Κροκος; crocus; crocus sativus.

Κρομμιον, κρομυον; cepa; allium cepa.

Κροτον; ricinus; ricinus communis.

Κροτονειδας; ricinus; *idem.*

Κυαμος; faba; *fruit des* vicia faba, vicia serratifolia.

Κυαμος αιγυπτιος; faba ægyptia; *fruit du* nymphæa nelumbo.

Κυαμος ελληνικος; faba græca; *fruit du* celtis australis?

Κυκλαμινον; cyclamen; cyclamen europæum *ou* C. aleppense.

Κυδωνια μηλα; cydonia mala; *fruits du* pyrus cydonia.

Κυμινον; cuminum; cuminum cymimum.

Κυμινον αιθιοπικον βασιλικον; cuminum æthiopicum; cuminum cymimum? pimpinella anisum. *Variet.*

Κυνορροδον; rosa canina; rosa canina.

Κυνος βατος; rubus caninus; rubus cyaneus.

Κυπαρισσος; cupressus, cyparissus; cupressus sempervirens.

Κυπειρος; cyperus; cyperus esculentus.

Κυτισος; cytisus; medicago arborea.

Λαγοπυρος; lagopyrus; trifolium arvense? gnaphalium dioïcum?

Λαδανον; ladanum; *gomme résine du* cistus cretica.

Λακαφθος; o; *ecorce du* laurus cinnamomum.

Λαπαθον; rumex; rumex patientia.

Λαπαθον αγριον; rumex agrestis; idem.

Λεπιδιον; lepidium; lepidium latifolium.

Λευκοιον; viola alba; hesperis matronalis.

Λευκοιον μελαν; viola nigra; idem, *variété rouge.*

Λιβανωτος; thus; *encens.*

Λιδιον; o; *écorce de grenade.*

Λινον; linum; linum usitatissimum.

Λινοζωστις; mercurialis; mercurialis annua.

Λωτος; lotus; rhamnus ziziphus, trigonella corniculata.

Λυγος; vitex; vitex agnus castus.

Μαλαχη; malva; malva rotundifolia.

Μαλαχη αγριε; malva sylvestris; malva sylvestris.

Μανδραγορας; mandragoras; atropa frutescens? atropa mandragora? actæa spicata.

Μαννα; pollen, manna thuris; o.

Μαννωδες; o; idem.

Μαραθρον; fœniculum; anethum fœniculum.

Μηκων; papaver, peplum; papaver somniferum.

Μηκωνιον; peplus, peplum, meconium; euphorbia peplus; *opium ou suc du* papaver somniferum.

Μηλα; mala; *fruits du* pyrus malus.

Μηλα αγρια; mala sylvestria; *fruits du* pyrus malus *à l'état sauvage.*

Μελανθιον; melanthium; nigella arvensis.

Μελανθιον κυπριον; o; nigella damascæna.

Μελια; fraxinus; fraxinus excelsior.

Μελικεδρινον; mel cedrinum; *suc mielleux du* pinus cedrus.

Μελιλωτος; sertula campana, melilotus; trifolium melilotus.

Μηλον; *voyez* μηλα.

Μεσπιλον et μεσπιλος; mespilus; mespilus germanica.

Μινθη; mentha; thymus mastichina, tanacetum balsamita, balsamita suaveolens.

Μινθη χλωρα; o; mentha viridis.

Μωδον, μωδος; o; bryonia alba.

Μορα; mora; *fruits du* morus nigra?

Μορα βατων; *fruits du* rubus fructicosa.

Μυκητες; fungi; boletus igniarius; *et autres champignons.*

Μυρικη; myrica; tamarix orientalis.

Μυρρινη αγρια; myrtus agrestis; ruscus aculeatus.

Μυρσινη; myrtus; myrtus communis.

Μυρτιδανον; myrtidanum; *excroissance du* myrtus communis.

Μυρτος; myrtus, myrtillus; myrtus communis.

Μυρτος μελανος; myrtus nigra; vaccinium myrtillus.

Ναπυ; *voyez* σιναπι.

Ναρκισσος; narcissus; narcissus poëticus.

Ναρδον ou ναρδος; nardus; valeriana nardus.

Ναρθηξ; ferula; ferula communis.

Ξανθιον; xanthium; xanthium spinosum?

Οχρος; ervilia; pisum ochrus.

Οκιμον; ocimum; amaranthus oleraceus, ocimum gratissimum.

Οινανθη; œnanthe; saxifraga cotyledon.

Οινανθη αμπελικη; œnanthe vitium; vitis sylvestris, TOURNEFORT.

Ολεια; olea; olea europæa.

Ολυνθος; grossus; *fruit du* ficus carica.

Ολοκονιτις; o; bunium bulbocastanum?

Ομφαλαξ λευκη; uva acerba alba; *verjus* ou *fruits verts du* vitis vinifera.

Ονοβληχον; o; saxifraga cotyledon.

Οφις; serpens; thymus serpillum?

Οποβαλσαμον; opobalsamum; *baume de l'*amyris opobalsamum.

Οριγανον; origanum; origanum heracleoticum.

Οριγανον κεφαλωδες; origanum capitatum; origanum vulgare.

Ορμινον; horminum; salvia sclarea.

Οροβος; orobus, ervum; orobus albus, O. canescens.

Ουα; sorba; sorba, *fruits du* sorbus domestica.

Πανακη; panax, panacea; pastinaca opopanax.

Παρθηνιον; parthenium; matricaria parthenium; anthemis cotula, anthemis arvensis.

Πεγανον; ruta; ruta graveolens.

Πελεκινος; pelecinum; coronilla securidaca.

Πενταφυλλον λευκον; quinquefolium album; potentilla nitida.

Πενταφυλλον μελαν; quinquefolium nigrum; potentilla reptans.

Πεπερι; piper; *fruits du* piper nigrum.

Πεπλιον; peplium; euphorbia peplus.

Πεπλος; peplum; euphorbia peplis.

Περιστερεον; verbena, herba columbaris; verbena officinalis.

Περσεη; persea; laurus persea.

Πευκεδανον; peucedanum; peucedanum officinale.

Πισσος; pisum; lathyrus sativus.

Πιτυς; pinus; pinus sylvestris.

Ποιη; herba; potentilla argentina: tormentilla erecta.

Πολιον; polium; teucrium polium.

Πολυκαρπον; cratæogonon; polygonum aviculare.

Πολυποδιον; polypodium; polypodium vulgare.

Πολυκνεμον; polycnemum; tanacetum vulgare.

Πρασιον; marrubium; marrubium vulgare.

Πρασον; porrum; allium porrum.

Πρινος; ilex; quercus coccifera.

Προμαλον; promalum; prunus spinosa?

Πυρος; triticum; triticum æstivum?

Πυρος σητανιος; triticum setanium; triticum hybernum?

Πυρος τριμηναιος; triticum trimestre; triticum trimestre.

Πυξος; buxus; buxus sempervirens.

Ραμνος; rhamnus; lycium europæum.

Ραφανις; raphanus; raphanus sativus.

Ριζα; radix; *racines des* raphanus sativus: momordica elaterium.

Ριζα αιθιοπικα; radix æthiopica; *racine du* ferula assafœtida.

Ριζα λευκη; radix alba; *racine de l'*arum dracunculus.

Ριζα μελαινα; radix nigra; *racine de l'*helleborus orientalis?

Ροα ou ροια; malus punica, malum punicum; *arbre et fruit du* punica granatum.

Ροδον, ροδος; rosa damascæna *et* rosa centifolia.

Ροη, ροος; rhus, fluentum; rhus coriaria.

Ροια; *voyez* ροα.

Σαγαπηνον; sagapenum; *gomme résine* sagapenum.

Σανδαρακη; sandaracum; *résine du* juniperus oxycedrus.

Σαυριδιον; nasturtium; lepidium sativum.

Σκαμμονια, σκαμμονιη; scamonia; *gomme résine du* convolvulus scammonia.

Σχεδιας μικρα; anchusa parva; anchusa angustifolia.

Σχεδιας μεγαλα; anchusa magna; anchusa officinalis.

Σχινος; lentiscus; pistacia lentiscus.

Σχοινος σχοινος ευοδες; juncus oduratus; andropogon schænanthus.

Σκιλλα; scilla; scilla maritima.

Σκολοπενδριον; scolopendrium; asplenium scolopendrium.

Σκοροδον; allium: allium sativum.

Σκυλλα; *voyez* σκιλλα.

Σελινον; apium; apium petroselinum.

Σελινον ελειον; apium palustre; apium graveolens.

Σεμιδαλις; similago; *fleur de farine de froment.*

Σεσαμοιδης: sesamoïdes; isopyrum thalictroïdes *et* isopyrum aquilegioïdes.

Σεσαμον; sesamum; sesamum orientale.

Σεσελι; seseli; ligusticum peloponesiacum.

Σεσελι μασσιλιοτικον; seseli massiliense; seseli tortuosum.

Σικυα; cucurbita; cucurbita pepo.

Σικυος; cucumis; cucumis sativus.

Σικυος αγριος; cucumis sylvestris; momordica elaterium.

Σικυονη μακρη; cucurbita longa; cucurbita lagenaria.

Σιδιον; malicorium; *écorce de grenade.*

Σιλφιον; laserpitium, laser; *gomme résine du* ferula assafœtida.

Σιναπι; sinapi; sinapis nigra, S. orientalis.

Σιον, sium; sisymbrium aquaticum? sium græcum?

Σισυμβριον; sisymbrium; thymus vulgaris.

Σταφυλινος; staphylinum; daucus carota.

Σταφυσαγρια; staphysagria; delphinium staphysagria.

Σταφυς; uva passa; *fruits secs du* vitis vinifera.

Στελις, στελεχος; stelis; loranthus stelis.

Στοιβη; stœbe; poterium spinosum.

Στρουθιον; struthium; saponaria officinalis, S. vaccaria, *ou* S. orientalis.

Στρυγις; *voyez* τρυγη.

Στρυχνος; solanum; solanum nigrum.

Στυβη; *voyez* στοιβη.

Στυραξ; styrax; *baume du* styrax officinalis.

Συκα; ficus; *fruits du* ficus caryca.

Συκαμινος; morus arbor; morus nigra.

Συκη; ficus; ficus caryca.

Τελεφιον; telephium; sedum telephium.

Τελις ou Θελις; fœnum græcum; trigonella fœnum græcum.

Τερμινθος; terebinthus; pistacia terebinthus.

Τευτλιον, τευτλον; beta; beta cicla, Murray.

Τευτλιον λιπαρον; beta pinguis; beta vulgaris.

Τιθυμαλλον, τιθυμαλος; tithymalus; euphorbia verrucosa, E. serrata.

Τιθυμαλλον μεγαλον; tithymalus magnus; euphorbia characias.

Τραγιον; tragium; hypericum hircinum.

Τριβολος θαλασσιος; tribulus mari adjacens; echinophorus spinosa.

Τριγη; tragus; hordeum zeocriton.

Τριφυλλον; trifolium; psoralea bituminosa.

Υοσκυαμος; hyoscyamus; hyosciamus niger.

Υπερικον; hypericum; hypericum perforatum.

Υποκιστις; hypocistis; cytinus hypocistis.

Υσσωπον; hyssopus; origanum smyrnium?

Φακος; lens, lenticula; ervum lens.

Φακος ευωδης; lenticula odorata; ervum ervilia.

Φηγος; fagus; quercus esculus.

Φιλιστιον; aparine, philistion; galium aparine? xanthium strumosum?

Φλομος; verbascum; phlomis laciniata.

Φοινικικος κοκκος; granum purpureum; *gallinsecte du* quercus coccifera.

Φοινικοβαλανον; palmula; *fruit du* phœnix dactilifera.

Φραγμιτης; arundo; arundo phragmites.

Φυλλον λιβυκον; folium libycum; *semence du* ferula assa-fœtida.

Χαλβανον; galbanum; *suc du* bubon galbanum.

Χαμαιλεων; chamæleon; centaurea crocodilium, carthamus corymbosus.

Χαριεν; o; cyclamen alepense?

Χονδρος; alica; *farine du* triticum spelta.

Ψευδοδικταμνον; pseudodictamnus; marrubium pseudodictamnus.

Ψιλωθριον; vitis sylvestris; humulus lupulus.

Ψωρα, ψωρα αγριελαιης; scabies, scabies; scabies oleastri; lichen caperatus, L. prunastri.

